# Supplementary figures and images for: Systematic analysis of Plasmodium myosins reveals differential expression, localisation, and function in invasive and proliferative parasite stages
Source: Cell Microbiol. 2019 Jul 23;21(10):e13082. doi: 10.1111/cmi.13082 (PMC6851706; doi:10.1111/cmi.13082)

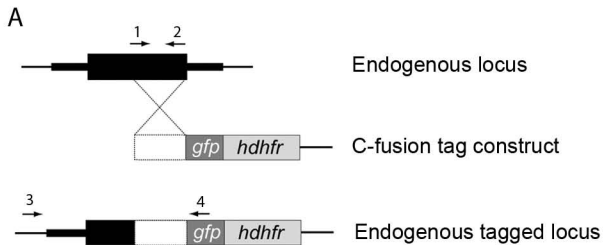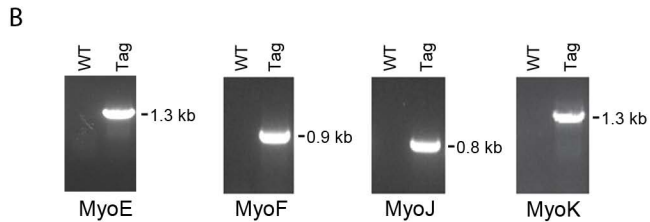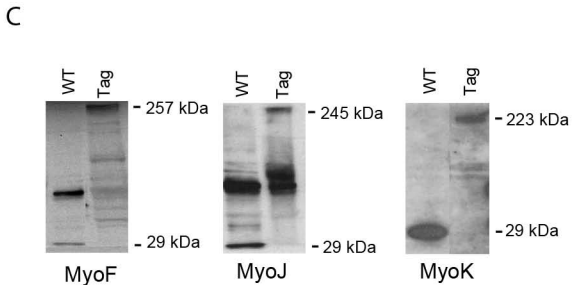

**D**

|          | Molecular weight | Unique peptide | Protein coverage |
|----------|------------------|----------------|------------------|
| MyoE-GFP | 228 kDa          | 67             | 41%              |

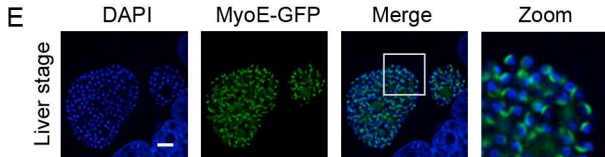

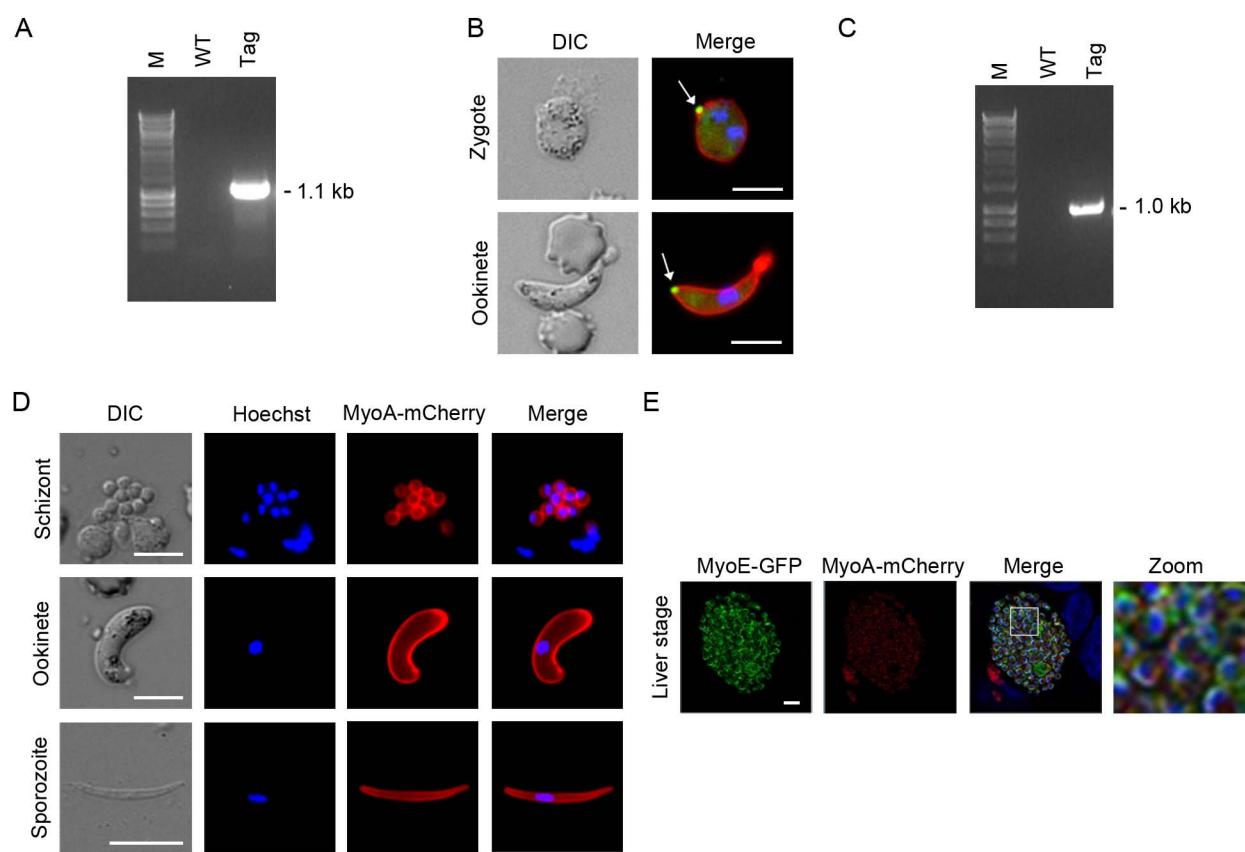

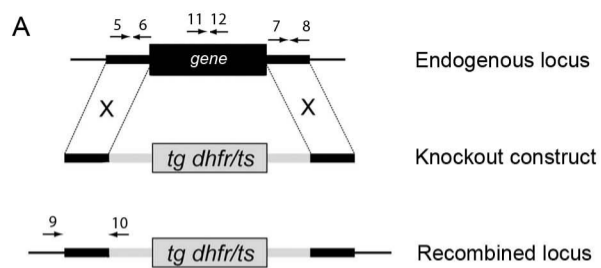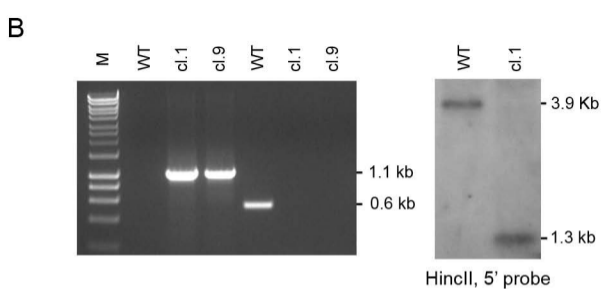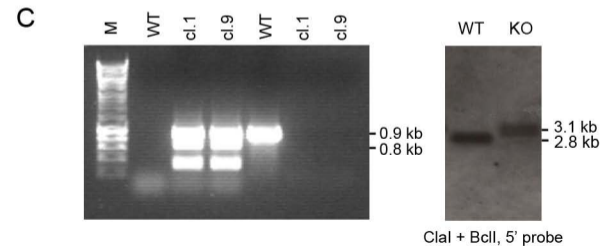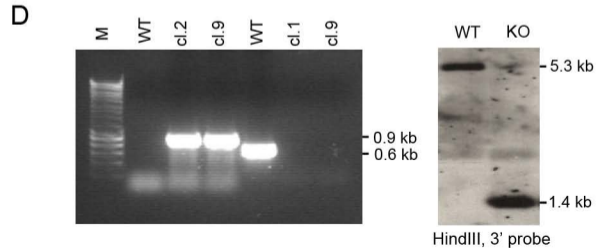

Supplement: Supplementary file 7 — Figure S1: Myosin tag design – (A) Schematic for C‐terminal GFP tagging of all the myosins by single crossover integration of the gfp and human dhfr selectable marker at the 3’ end of the gene. The 3’ region of the myosin CDS, immediately upstream of the stop codon, was amplified using primers 1 and 2. (B) PCR analysis to confirm integration for the four myosin tags generated in this project, by amplification of DNA with primers 3 and 4. (C) Western blot analysis of MyoJ‐GFP (oocyst) MyoK‐GFP (activated gametocytes) and MyoF‐GFP (asexual stage) generated in this project, and compared with WT‐GFP (29 kDa) using a GFP‐specific antibody. (D) Immunoprecipitation of MyoE‐GFP with a GFP‐specific antibody followed by tryptic digestion and mass spectroscopy identified 67 unique peptides covering 41% of the MyoE protein sequence. (E) Liver stage expression of MyoE‐GFP, showing DAPI (blue), GFP (green) and merge (DAPI and GFP) images. The zoom panel is a higher magnification display of the area enclosed by the white box in the merge image. Scale bar = 5 μm. Figure S2: Additional tag validation – (A) Integration PCR analysis of MLC‐B‐GFP parasite line – based on the same strategy as shown in Figure S1A – using primers 3 and 4. (B) MLC‐B expression in both young (<2 hr) retort and ookinete. DIC images are on the left, and on the right are merged: Hoechst 33342 (blue), GFP (green) and 13.1 (red), a cy3‐conjugated antibody. White arrow indicates the location of MLC‐B protein. (C) Integration PCR validation of MyoA‐mCherry parasite line – based on the same strategy as shown in Figure S1A – using primers 3 and 4. (D) Expression of MyoA‐mCherry in schizonts, ookinetes and sporozoites (Green et al., 2017). The panels from left to right are DIC, Hoechst 33342 (blue), MyoA‐mCherry (red), and merged blue and red. (E) Expression of MyoE‐GFP (green), MyoA‐mCherry (red) in the liver stage and a merged image including Hoechst stained DNA. A higher magnification image (zoom) is shown. Scale [file CMI-21-na-s007.pdf]
